# Beyond Seed and Soil: A Bacterial-Passenger Framework for Organ-Tropic Breast Cancer Metastasis

**DOI:** 10.34133/research.1429

**Published:** 2026-09-23

**Authors:** Yumian Huang, Zhiwei Li, Fu Gao, Lei Huang, Gang Sun

**Affiliations:** ^1^ Xinjiang Key Laboratory of Tumor Microenvironment Homeostasis Regulation and Targeted Intervention, Urumqi, Xinjiang, China.; ^2^ Precision Medicine Center, People’s Hospital of Xinjiang Uygur Autonomous Region, Urumqi, Xinjiang, China.; ^3^ Department of Surgery, Yale School of Medicine, New Haven, CT, USA.; ^4^ Department of Molecular Cell and Cancer Biology, University of Massachusetts Chan Medical School, Worcester, MA, USA.; ^5^ Department of Breast and Thyroid Surgery, People’s Hospital of Xinjiang Uygur Autonomous Region, Urumqi, Xinjiang, China.

## Abstract

Breast cancer metastasis reflects both tumor cell heterogeneity and compatibility with distant organ microenvironments. Tumor-resident bacteria add a potential nongenetic layer to this process, but the strength of evidence varies substantially across proposed mechanisms. Here, we introduce the concept of a “bacterial passenger”, which is operationally defined as a viable bacterium localized within, rather than merely adherent to, a tumor cell or circulating tumor cell during dissemination. Passenger status must be distinguished from nonviable microbial DNA, extracellular signals, and environmental or reagent contamination through viability-aware imaging, culture or functional assays, and matched strain-level evidence across primary tumors, circulating tumor cells, and metastases. The term describes physical codissemination and does not itself imply a causal effect. Breast cancer models show that intracellular bacteria can increase survival under fluid shear stress and metastatic colonization, yet direct evidence that they determine organ tropism, brain metastasis, or treatment resistance remains limited. We therefore present the seed–soil–passenger triad as a bacteria-centered, falsifiable extension of the seed–soil framework rather than a replacement. Its value lies in testable predictions about codissemination, organ-dependent effects, and stage-specific bacterial perturbation, coupled to rigorous low-biomass controls. Fungi and viruses are outside the present operational framework because comparable evidence for viable codissemination with breast cancer cells is not available.

## Introduction

**Fig. 1.**
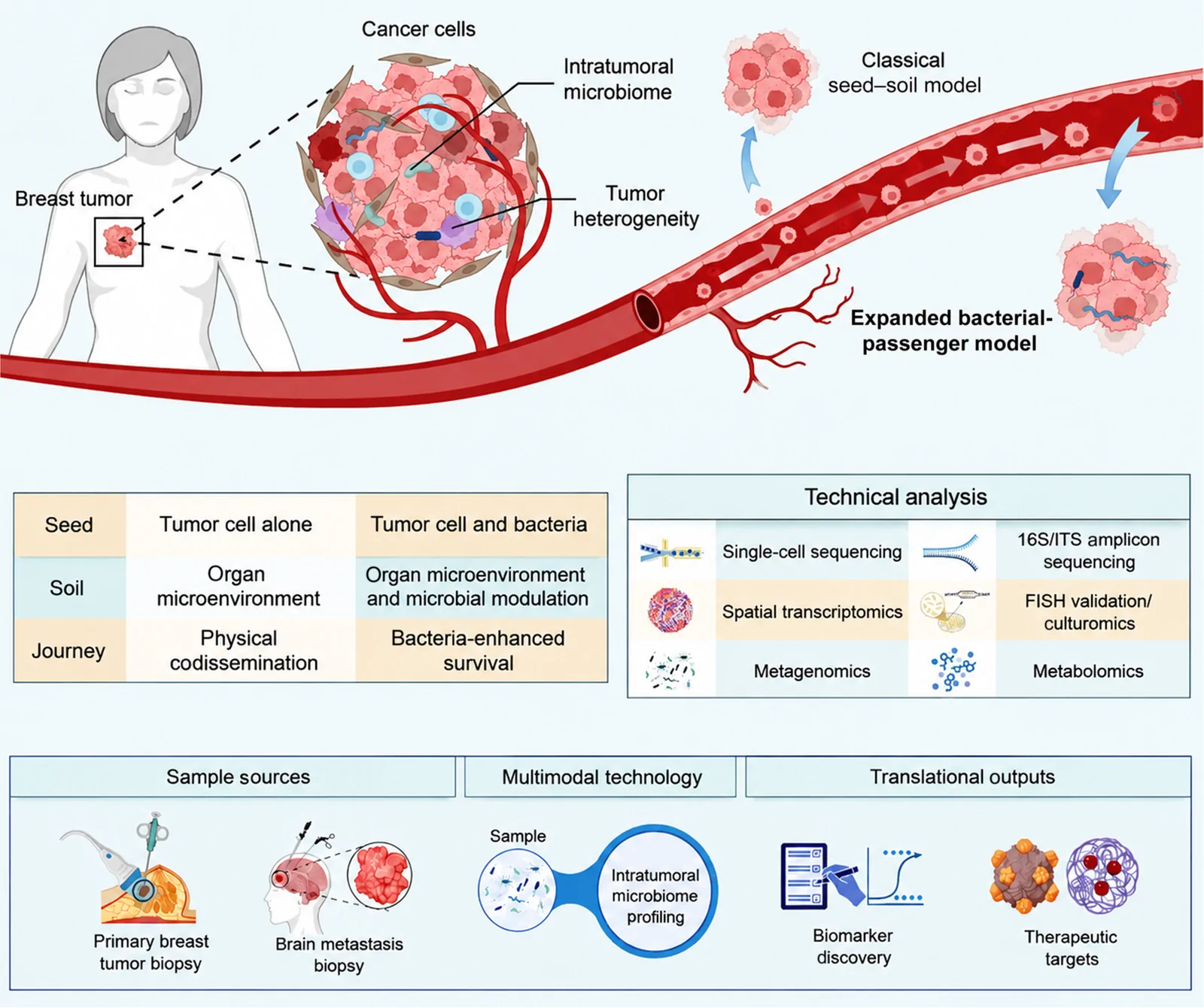
The proposed bacterial-passenger model extending the seed–soil paradigm of breast cancer metastasis. Intracellular bacteria may codisseminate with circulating tumor cells and influence metastatic fitness, with potential effects on organ-specific niche permissiveness, and host immune responses. Multi-omics profiling enables systematic characterization of these host–microbe interactions and their translational potential for precision oncology.

Approximately 2.3 million breast cancer cases were diagnosed worldwide in 2020 [1]. Surrogate clinicopathologic subtypes based on estrogen receptor, progesterone receptor, human epidermal growth factor receptor 2 (HER2), and Ki-67 are commonly used to approximate luminal, HER2-positive, and triple-negative disease [2]. Patterns of distant spread differ across these subtypes, although no subtype–organ association is absolute [3]. These patterns remain grounded in Paget’s seed–soil principle, in which disseminated tumor cells colonize organs whose vascular, stromal, immune, and metabolic conditions support their survival [4]. The framework has evolved well beyond a static tumor–organ pairing and already accommodates nongenetic and microenvironmental determinants (Fig. 1).

Tumor microbiome studies identify low-biomass, cancer-type-associated bacteria, often within tumor or immune cells [5,6]. A pan-cancer cohort also found organ-associated microbial distributions across metastatic biopsies, an association that does not demonstrate microbial control of destination or cocarriage with tumor cells [7]. In breast cancer models, intracellular bacteria carried by circulating tumor cells increased resistance to fluid shear stress and promoted metastatic colonization [8], while hematogenous *Fusobacterium nucleatum* colonized mammary tumors and increased metastatic progression in mice [9]. These findings establish that tumor-associated bacteria can affect dissemination; they do not by themselves establish a general microbial control of organ preference. Our proposed advance is therefore narrower than the claim that bacteria influence metastasis. We define a falsifiable passenger state that requires physical codissemination and persistence across the primary tumor–circulating tumor cell–metastasis axis and then ask whether that state changes metastatic success in an organ-dependent manner.

## A Bacteria-Centered, Evidence-Graded Model

Though intratumoral communities encompass bacteria, fungi, and viruses, available mechanistic evidence focuses largely on intracellular bacteria. We therefore center the operational model on bacteria because comparable evidence for viable fungi or viruses codisseminating with breast cancer cells is not available. Three evidence levels should be separated. First, human tumor studies support bacterial presence, spatial organization, persistence, or organ-associated patterns without establishing organotropic causality [6,7,10,11]. Second, breast cancer models support bacterial enhancement of dissemination or colonization [8,9]. Mechanisms reported in other tumors, including immune effects, require breast-specific testing [12,13]. Third, organ-specific niche regulation, treatment resistance, and microbial effects on the blood–brain barrier remain hypotheses.

Brain metastasis illustrates this boundary. Breast cancer cells rely on established tumor-intrinsic programs that contribute to crossing the brain endothelium and colonizing the brain [14]. Bacterial infection models show that microbes or their products can disrupt barrier integrity [15], but this does not demonstrate a tumor-associated bacterial mechanism in breast cancer brain metastasis. Direct evidence connecting intratumoral microbes to breast cancer brain metastasis remains scarce and requires rigorous experimental validation. Direct transport of viable bacteria within tumor cells, local release of microbial products or metabolites, systemic inflammation with endothelial injury, and glial immune modulation are distinct hypotheses and should not be collapsed into one established “microbial broker” pathway [14–16]. The model would be weakened if bacteria were detected only as cell-free DNA, if matched viable organisms were absent from circulating tumor cells and metastases, or if bacterial perturbation changed overall tumor burden without altering dissemination or organ-specific colonization.

## Testable Predictions and Methodological Priorities

The passenger model makes 3 discriminating predictions. First, viable strain-resolved bacteria should be shared among matched primary tumor cells, circulating tumor cells, and selected metastatic lesions more often than expected from blood, adjacent tissue, and laboratory controls. Second, depleting or disabling candidate bacteria after intravasation should reduce shear resistance or metastatic seeding, and same-strain rescue should restore the phenotype without requiring a change in primary tumor growth. Third, after controlling primary tumor burden and circulating tumor cell number, passenger perturbation should change the relative distribution of metastases among organs. A change only in total metastatic burden would not support an organ-tropism mechanism. Failure of these tests would weaken support for a passenger-mediated mechanism of organ tropism.

Because tumor samples contain little microbial biomass, contamination can dominate sequencing results [17]. Here, “microbiota” denotes living organisms or communities; “microbiome” includes their genes, products, and ecological context; and “microbial signal” denotes a sequence, antigen, or metabolite that does not by itself establish viability or localization. Required controls include sampling, extraction, reagent, and no-template blanks; positive or spike-in controls; randomized and batch-balanced processing; paired blood and adjacent tissue as biological comparators rather than blanks; batch-aware decontamination; orthogonal spatial localization; viability assessment; and explicit discrimination of intracellular, adherent, extracellular, and contaminant signals. Spatial tools such as MERFISH and 10x Visium map host gene expression patterns and spatial tumor organization but require dedicated microbial probes, strict validation, and contamination controls to localize microbes; they do not automatically provide robust microbial profiling [11]. Culturomics and targeted sequencing can complement imaging, but detection alone is insufficient to establish function [18].

Mechanisms shown in nonbreast settings, including TIGIT-dependent immune inhibition [13], are labeled as indirect evidence rather than breast cancer passenger mechanisms. A microbiome-informed biomarker would require prospective validation for its intended clinical use. A microbiome-targeted therapy would require strain- or function-specific causal evidence and demonstration of clinically relevant benefit without unacceptable disruption of beneficial microbiota.
